# Condom Use at Last Sexual Intercourse and Its Correlates among Males and Females Aged 15–49 Years in Nepal

**DOI:** 10.3390/ijerph15030535

**Published:** 2018-03-16

**Authors:** Bimala Sharma, Eun Woo Nam

**Affiliations:** 1 Yonsei Global Health Center, Yonsei University, Wonju 03722, Korea; bimalasharma@gmail.com; 2 Department of Health Administration, Graduate School, Yonsei University, Wonju 03722, Korea

**Keywords:** condom use, correlates, sociodemographic factors, gender, multiple sexual partners, HIV knowledge, Nepal

## Abstract

This study aimed to assess the prevalence and correlates of condom use at last sexual intercourse among people aged 15–49 years in Nepal. Secondary data analysis was performed using the Nepal Demographic and Health Survey 2011. The study was restricted to the respondents who reported ever having had sexual intercourse; 9843 females and 3017 males were included. Condom use was assessed by asking if respondents used condoms in their most recent sexual intercourse. Chi-square test and multivariate logistic regression analysis were performed using Complex Sample Analysis Procedure to adjust for sample weight and multistage sampling design. Overall, 7.6% of total, and 16.3% of males and 6.2% of females reported using condoms in their last sexual intercourse. Living in Far-Western region, age and wealth quintile were positively associated with condom use in both males and females. Being unmarried was the most important predictor of condom use among males. Higher education was associated with increased likelihood of condom use in females. However, mobility, having multiple sexual partners, and HIV knowledge were not significant correlates of condom use in both sexes. A big difference was observed in the variance accounted for males and females; indicating use of condoms is poorly predicted by the variables included in the study among females. Condom use was more associated with sociodemographic factors than with sexual behavior and HIV knowledge.

## 1. Introduction

The shift towards later marriage in most countries has led to an increase in premarital sex. The prevalence of condom use has increased almost everywhere, but rates remain low in many developing countries [1]. Condoms, one method of family planning (FP), provide substantial protection against human immunodeficiency virus (HIV) infection [2]. Although the South Asian Region still has a low prevalence of HIV, the highest number of people with HIV outside Africa resides in India, a bordering country of Nepal. The importance of maintaining the low prevalence status in the region cannot be overemphasized [3]. 

Previous studies found that condom use was associated with a large number of community factors such as type of residence [4,5], socio-demographic factors such as age and sex [5,6,7], marital status [8,9,10], education [4,9], occupation [6,9,11], and economic status [1,5,7]. Previous studies show mixed evidences regarding the relationship between multiple sexual partners and condom use [11,12]. Greater knowledge of sexually transmitted infections (STIs) was also found to be associated with increased likelihood of condom use during the last sexual encounter [11]. 

The potential for HIV infection is increasing with the changing social and economic environment in Nepal. Increasing internal and international migration of the population, modernization, and development of transportation and communication networks are creating more favorable environments for social interactions between people. This is providing the opportunity for pre-marital, extramarital, and unsafe sexual activity among them, thereby increasing their risk of acquiring HIV and unwanted pregnancies [13,14,15,16]. Different studies conducted among young people have reported that unsafe sexual behavior is prevalent and increasing [15,16,17,18]. In addition, extramarital sexual intercourse is also not uncommon among adult population in Nepal [19,20]. The vulnerability of HIV infection among low risk population such as wives is expected to increase, which might bridge the infection to the general population [21]. Condoms are effective in preventing both pregnancy and STI/HIV [22]. Condom use varies by marital status, with unmarried individuals more likely to use them while married couples tend to choose hormonal or permanent FP methods to prevent unwanted pregnancies [23]. However, as both populations, unmarried and married individuals may be at risk of STIs and HIV, promoting condom use among both groups is one of the recommended strategies for dual protection; and this was the basis for conceiving the study.

The FP program is one of the priority programs of the Nepalese government. The Ministry of Health distributes condoms free of charge through all health facilities, including outreach clinics and Female Community Health Volunteers. However, their utilization seems low as compared to other birth spacing methods in Nepal [24]. Global trend analyses of sexual behavior recommend that public health interventions should address the broader determinants of sexual behavior, such as gender, poverty, and mobility, in addition to individual behavioral change [1]. Thus, a conceptual framework for action on the social determinants of health of the World Health Organization was considered to examine the factors associated with condom use in the study. This model highlights the importance of socioeconomic factors as determinants of behaviors [25]. Existing efforts to address HIV/STI vulnerability and risk in the population focus primarily on risk-taking behavior, and largely fail to address contextual issues that create and facilitate risky behavior and situations in Nepal [26]. Although existing literature shows that unsafe sexual practices conducive to transmitting HIV infection are prevalent, there is limited evidence regarding condom use among the general population. This study aimed to assess the prevalence and correlates of condom use at last sexual intercourse among males and females aged 15–49 years in Nepal.

## 2. Methods

### 2.1. Study Area, Study Design, and Sampling

Nepal is a developing country in Southeast Asia with a human development index of 0.548, and a life expectancy of 69.6 years [27]. Geographically, the country is divided into three ecological belts: the Northern Range Mountain, the Mid-range Hill, and the Southern Range Terai (flat land). An analytical, cross-sectional study was conducted from the secondary data of the Nepal Demographic and Health Survey (NDHS) 2011. A publicly available dataset was obtained from the MEASURE DHS website [28]. The dataset was created by merging relevant information from the women’s questionnaire and the men’s questionnaire. The details of the questionnaire and procedures can be found in the website and survey report [28,29].

The NDHS used a multistage cluster sampling procedure for data collection. In the first stage, a total of 95 urban and 194 rural enumeration areas (wards in the village development committees and sub wards in the municipalities) were selected using a proportionate probability sampling method. In the second stage, households within each enumeration area (EA) were selected using systematic sampling technique. In this stage, 35 households in each urban EA and 40 households in each rural EA were randomly selected. The NDHS 2011 completed a survey of 12,674 females and 4121 males. The study was restricted to the respondents who reported ever having had a sexual encounter in the past; 9843 females and 3017 males were selected for the analysis. Therefore, total sample size for the study was 12,860 individuals.

### 2.2. Measurement of Variables

#### 2.2.1. Condom Use

Condom use was measured by the question, “Was a condom used the last time you had sexual intercourse?” The responses were categorized as (i) “yes = 1” and (ii) “no= 0” for the analysis, as measured in previous studies [6,8,11].

#### 2.2.2. Explanatory Variables

The explanatory variables are shown in Table 1. Ethnicity was categorized into three groups: upper caste (Hill Brahmin, Hill Chhetri, Terai Brahmin, and Terai Chhetri), lower caste (Hill Dalit and Terai Dalit), and others (all other recorded ethnicities) [30]. Economic status was evaluated by NDHS 2011 using principal component analysis of more than 40 assets as variables [31]. The calculated score was divided into 5 quintiles and provided in the NDHS dataset ranging from poorest to richest. Most of the other variables were categorized as they were measured by the NDHS 2011 [29]. HIV knowledge was measured using ten relevant questions selected from the NDHS survey questionnaire (Table A1). Correct responses were coded as “1” and incorrect or uncertain responses were coded as “0”. The items were summed to create an HIV knowledge score, with higher scores indicating more knowledge about HIV transmission and prevention [32].

### 2.3. Data Analysis Methods

The data were analyzed using SPSS version 24.0 (IBM Corp., Armonk, NY, USA). Descriptive statistics were used to describe the characteristics of the study population and the prevalence of condom use. The chi-square (χ^2^) test was used to analyze the association between the explanatory variables and condom use. Multivariable logistic regression analysis was conducted in a hierarchical order adapting the concept from a previous study using the NDHS 2011 [33]. Model 1 comprised community factors, model 2 included the factors of model 1 and sociodemographic factors, model 3 included the factors of model 2 and behavioral factors, and model 4 comprised the factors of model 3 and HIV knowledge. The analyses were conducted separately for males and females. Adjusted odds ratios (AORs), 95% confidence intervals (CIs), and p values were presented. The level of significance was set at 5%. Based on the sample weights, strata, and cluster given in the survey dataset, a Complex Sampling Plan File was prepared. All the analyses were performed using the Complex Sample Analysis procedure, which is recommended to adjust for sample weight and multistage sampling using the DHS data [34].

### 2.4. Ethical Approval

The NDHS 2011 was approved by the Nepal Health Research Council and Ethical Review Board of ICF Macro International. The dataset is completely anonymous and distributed in the public domain. Therefore, an independent ethical approval was not required.

## 3. Results

Of a total of 12,860 respondents, 76.6% were females, 19.1% were in the age group of 25–29 years, and 93.9% respondents were married. Of the total number of participants, 41.1% had no formal schooling, 52.6% were involved in agriculture, and 17.0% respondents were from the poorest groups. In the study, 34.5% of the respondents were from the upper caste, and 84.6% were Hindus. The majority of the respondents were from the Terai region; 33.9% respondents were from Central development region; and 86.0% were from rural areas. In the last 12 months, 14.0% of the total respondents reported being away from home for more than one month. Of the total respondents, 12.3% had multiple sexual partners in their lifetime. Lifetime multiple sexual partnership was 28.7% among currently unmarried and 11.2% among married. The mean HIV knowledge score was 7.5 out of a maximum of 10. The mean age at first sex was 17.8 years. During their last sexual intercourse, 7.6% of the respondents, 16.3% of males, and 6.2% of females used condoms with their partners (Table 1).

Table 2 shows the proportions of condom use by explanatory variables. Condom use at last sexual intercourse was more than double among males than among females (16.3% vs. 6.2%). The percentage of people using condoms was found decreased along with the increase in age, ranging from 14.0% in the age group of 15–24 years to 4% in the age group of 40–49 years. The highest proportion of condom use (11.3%) was found in the upper caste and the lowest (4.2%) in the lower caste. Condom use percentage was almost similar across different religious groups ranging from 7.6% among other religious group to 8.9% among Hindus. The percentage of people who used condoms at last sexual episode was 62.3% among respondents currently unmarried, and 7.3% among those married. The frequency of condom use was 3.2% in the no schooling group and 24.3% among the respondents with higher education. Similarly, the proportion of condom use was lowest (5.5%) among agricultural workers and the highest (15.8%) among service holders. Use of condoms was 4% in the poorest and 16.1% in the richest wealth quintile. The highest proportion of condom use (10%) was found in the Hill region, while the lowest (5.9%) in the Mountain region. Regarding the development region, condom use percentage was highest (14.0%) in the Far-Western region and the lowest (7.3%) in the Central region. Condom use was 15.0% among the individuals from urban area and 7.7% from rural area. The frequency of condom use was double among participants with multiple sexual partners than among those who did not have (14.6% vs. 7.9%).

Among the currently unmarried sample also, more males used condoms than females (68.3% vs. 20.9%). Use of condoms at last sexual intercourse was 69.1% among respondents of 15 to 24 years where as it was 0% among those who were in the age group of 40 to 49 years. Similarly, condom use frequency was 70.7% among respondents with higher education where as it was 19.8% among those who did not have formal education. The proportion of condom use was lowest (46.0%) among agricultural workers and highest (74.4%) among service holders. Condom use percentage was highest (80.3%) among the respondents belonging to richer group and the lowest (39.5%) among those who were from the poorest group. Ethnicity, religion, place of residence (by region and type), mobility, having multiple sexual partners did not have significant association with condom use among currently unmarried respondents.

Table 3 shows the logistic regression analysis of factors associated with condom use among males. In model 1, all three variables: ecological region, development region, and residence were significantly associated with condom use. In model 2, development region, age group, marital status, education, and wealth quintile were significantly associated with it. In model 3, all significant variables in model 2 except education and age at first sexual intercourse were significant. In model 4, all significant variables in model 3 remained significant, and HIV knowledge did not have any significant effect on condom use. Table 4 shows the logistic regression analysis of factors associated with condom use among females. In model 1, all three variables were significantly associated with condom use. In model 2, ecological region, development region, age group, ethnicity, marital status, education, and wealth quintile were significantly associated with it. In model 3, all significant variables in model 2 except ethnicity and marital status were significant. In model 4, all significant variables in model 3 remained significant, and HIV knowledge did not have any significant effect on condom use. 

In the adjusted analysis (model 4), as compared to the Eastern region, males living in the Far-Western region and females living in the Mid-Western and the Far-Western region had increased probability of using condoms. Older age group was statistically associated with lower use of condoms in both sexes, but the association was significant only for those belonging to 40–49 years as compared to the respondents of 15–24 years among females. Occupation and religion were not associated with condom use in both males and females. However, belonging to higher wealth quintile was associated with higher condom use in both sexes. Males belonging to richer and richest group were 110% and 225%, respectively, more likely to use condoms as compared to the poorest males. Females belonging to poorer, middle, richer, and richest group were 99%, 119%, 183%, and 383%, respectively, more likely to use them as compared to the poorest females. Mobility, multiple sexual partners, and HIV knowledge did not have association with use of condom in both sexes (Table 3 and Table 4).

Unmarried males were more likely (AOR, 12.8; CI, 8.2–20.0) to use condoms in their last sexual intercourse as compared to married males. Similarly, higher age at first sexual intercourse was associated with higher use of condoms among them (Table 3).

Females from the upper caste were more likely to use condoms than the females of the lower caste. Similarly, education was significantly associated with condom use only in females. Females belonging to primary and secondary education were 66% and 157%, respectively, more likely to use condoms in their last sexual intercourse as compared to the females from no formal schooling (Table 4).

The Cox and Snell *R^2^* was 1.8% in model 1, 19.1% in model 2, 21.1% in model 3, and 22.0% in model 4 among males (Table 3). Similarly, Cox and Snell R^2^ values of 1.2%, 5.3%, 5.5%, and 5.4% were obtained in model 1, model 2, model 3, and model 4, respectively, among females (Table 4). These meant that 22.0% and 5.4% variation in the frequency of condom use was determined by the factors of model 4 among males and females, respectively, in Nepal. It shows that condom use is mostly affected by sociodemographic factors than behavioral factors and HIV knowledge. In addition, the variation of the Cox and Snell *R*^2^ values among males and females shows that, unlike among males, condom use among females was less likely to be determined by the factors included in the models.

## 4. Discussion

The study identified the prevalence of condom use at last sexual intercourse and associated factors among 15–49 years male and female population in Nepal. A low prevalence of condom use at last sexual intercourse was found, and sociodemographic factors were significantly associated with condom use among a large sample of general population. Being male, being unmarried, and being young were most important. Unlike this study, most of the previous studies on condom use in Nepal dealt with particular type of people, especially high-risk population for HIV and among small samples.

The prevalence of condom use was further low among females as compared to males in the study. We found a shortage of evidence on condom use prevalence among the general population in Nepal. Small scale studies show low and irregular use of condoms even among casual partners [15,16,17,35]. In India, 4.8% of ever married women of 15–49 years reported condom use at their last sexual intercourse; and condom use was 32% with husband and 38% with boyfriend among young urban women [6,36]. As condoms are recognized as birth control, married heterosexual people may be reluctant to use them for HIV prevention. Although family planning services have emphasized condom use, this method represented only 3.5% in total contraceptive prevalence rate in Nepal. Use of other FP methods such as hormonal contraceptives and sterilization are most popular among women in Nepal [24]. In Nepal, men who reported a desire to have no more children were more likely to choose permanent methods [37]. Couples in which either husband or wife have been sterilized or women using other hormonal methods usually do not consider using condoms [23]. Therefore, low use of condoms might be because most of the participants were married and condoms are less likely to be used in such relationships, that they are not even considered as methods of family planning. Low perceived susceptibility for HIV infection and low felt need for FP could be why condoms were not used. 

The chi-square test showed that males were more likely to report condom use during their most recent sexual intercourse. The gender difference of condom use exists in total as well as unmarried samples in the study. Thus, we conducted gender-disaggregated analysis to find out correlates of condom use. There is a gender difference around sex, with women having fewer opportunities and less freedom than young men in Nepal [38]. This situation affects women’s possibility of asking for and using condoms with partners. A similar result was found in a previous study, where gender played a significant role in decision making in condom use; men were more likely to take decisions on condom use than women [39]. A study among female sex workers reports that violence from partners, resistance from partners, and lack of negotiation capacity were the important reasons for non-use of condoms with husbands and clients [40]. Condom use is with linked with commercial sex work; if a woman suggests or insists that her husband use condoms, he may believe that she suspects him of having a STD or being HIV positive [23]. Thus, it seems more unlikely to suggest or insist condom use with husbands and regular partners. Greater emphasis must be given to addressing the gender discrimination embedded in Nepalese culture to vulnerability to HIV/STI infection [26]. In the study, difference in the variance accounted for males and females in models notably reflects the issue related to gender power relation; perhaps the most important determinant of condom use among women might be their partners’ interest or decision than the factors included in the study. 

Older age was associated with a lower frequency of condom use in the study. Age is linked with awareness, marital status, and opportunity of having multiple sexual partners, which might influence condom use. Marital status was one of the most important determinants of condom use at last sexual episode among males. Condoms are more preferred with casual sexual partners than with a regular sex partner [10,23]. However, marital status did not influence condom use among females. The global trend shows that married women find negotiation of safer sex and use of condoms for FP more difficult than do single women [1]. A low proportion of unmarried females in the study might have influenced the association. In India, most married women have societal pressure to prove their fertility and they might not see any reason to use condoms as contraception. In addition, women may not consider condoms because they believe condoms interfere with their efforts to establish their relation with husbands or partners [23]. Likewise, a study in South Africa found widespread disapproval of condom use within marriage [4]. Type of sexual partner was the strongest predictor of condom use; it was higher among men who reported last sex with a casual partner [10]. It shows condom use is linked with casual sex and not considered within marital union. The misconception and reasons of non-use of condoms in marital relation need to be explored and addressed in Nepal.

Living in an urban or rural area did not influence use of condoms; rather there might be other factors which determine their use or non-use. However, development region had a significant effect on condom use in both sexes and ecological region among females. It indicates that some administrative regions and ecological regions are better for using condoms. In Nepal, a large number of health indicators are better in urban areas, and Central and Western development regions [23,25], but this was not true for condom use. As compared to the Eastern region, males living in Far-Western, and females living in Far-Western and Mid-Western regions had a significantly higher likelihood of condom use. The Far-Western region is considered as the most vulnerable region for HIV due to labor migration to India. The disease is commonly known as Mumbai disease in the region, because labor migrants who returned from Mumbai, a city of India, carry the infection with them [41,42]. Higher condom use in the region is a positive finding, but it is not sufficient to prevent new HIV infection [43]. 

The majority of the people in Nepal are Hindus. However, religion did affect use of condoms among males and female. Regarding ethnicity, condom use was significantly higher among the upper caste females as compared to the lower caste females. However, ethnicity did not have any effect on condom use among males. In general, use of condoms was not affected by religious as well as caste affiliation in Nepal. The condom promotion program should equally focus across people from all religion and ethnicity.

This study revealed that education did not have any significant association with condom use among males; but there was a significant association among females. This shows that formally educated women might have better negotiation power and skill for condom use with their partners. Higher education had higher odds of condom use in previous studies in different study settings [5,10]. Although there was a statistically significant association between education and condom use, total variance predicted by the analysis models was very low in females. This clearly shows that there are other factors not included in the study that determine use of condoms among females. The probability of condom use was higher among respondents belonging to poorer, middle, richer, and the richest groups as compared to the poorest group. Higher economic status might be associated with other factors such as awareness level and affordability of condoms at the time of need. Although condoms are provided free of charge through all government health facilities in Nepal [24], economic status might be associated with the capacity to afford condoms from private pharmacies due to issues regarding quality, confidentiality, or getting them at the time of need. Low condom use was also observed in the poor and middle wealth quintiles in a previous study from other countries [5]. However, use of condoms was not affected by the type of job people did in the study. Some studies in other countries show that occupation was correlated with condom use [9,11]. Thus, condom promotion should focus more on people of poor socioeconomic status.

In Nepal, both internal and international migration has been increasing in the last 2 decades; this is linked to the possibility of HIV transmission. In a study among returnee labor migrants from overseas in Nepal, 49% of respondents had sex with paid/unpaid partners, and only 61% used condom always [13]. The current study shows mobility was not significantly associated with condom use in both sexes in the adjusted analysis. This indicates increasing vulnerability of HIV transmission to the general population. In addition, both the married and unmarried population had had multiple sexual partners in their lifetimes. In the multivariable analysis, having multiple sexual partners was not statistically associated with condom use among both males and females. In Nepal, condoms are not usually considered among married couples. As most of the respondents were married, the last sexual intercourse might not have been with a casual partner. This might be one reason of insignificant association. However, the chi-square test shows having multiple sexual partners was also not significant with condom use among unmarried respondents. This shows both married and unmarried people are at risk of HIV transmission in Nepal. Older age at first sex was associated with increased likelihood of reporting condom use among male respondents. A study conducted in Botswana also reported that age at first sex was positively correlated to condom use for both males and females [9]. This might indicate responsible sexual behavior among those whose sexual debut was delayed. 

It is supposed that use of condoms is increased along with an increase in HIV related knowledge among people. However, higher HIV knowledge was also not associated with condom use in both sexes in the study. In a study conducted in India, knowledge on HIV prevention was significantly associated with condom use during last sex with husband [6]. Greater knowledge of STIs was also found to be associated with increased likelihood of condom use during the last sexual encounter in Jamaica [11]. Despite a high level of knowledge, reported condom use was very low among young migrant factory workers in Nepal, as reported in a previous study [15]. This finding may indicate that providing knowledge about HIV infection alone is not sufficient to promote condom use in Nepal. 

### 4.1. Limitation of the Study

The study is based on the large national survey conducted using standard questionnaire and survey procedure. The study still has some limitations. First, the survey type may induce behavioral desirability bias. Individuals may be reticent or embarrassed to express their real sexual behavior. It is challenging to validate the respondents’ answers. Second, due to cross-sectional nature of the survey, cause and effect relationships could not be established. Third, as there was limited literature, some references are compared and discussed from non-similar settings in spite of different HIV risk and contexts.

### 4.2. Implication of the Study

Gender, age, marital status, education, economic status, and place of residence are important factors associated with condom use among general population of Nepal. The study also shows that condom use is mostly affected by sociodemographic factors than multiple sexual partnership and HIV knowledge. Thus, condom promotion is a multi-dimensional issue. The government of Nepal should consider these social determinants to promote condom use for dual protections: unwanted births and HIV infection. The Ministry of Health should collaborate with other social sectors such as gender development, education, finance, and local development etc. In addition, the difference in variance accounted for males and females shows that condom use among females is mostly affected by the factors other than included in the study models; perhaps it would be their partner’s willingness and decision. Further studies, especially qualitative studies are required to explore reasons of non-use of condoms among females.

## 5. Conclusions

The prevalence of condom use at last sexual intercourse was low in Nepal. Living in the Far-Western region, younger age, upper caste, and belonging to a higher wealth quintile were significantly associated with the increased likelihood of condom use in both sexes. However, religion, occupation, and residence type were not significant correlates of condom use. Similarly, HIV knowledge, having multiple sexual partners, and mobility also did not have significant association with condom use in both males and females. Being currently unmarried was the most important predictor of using condoms at most recent sexual encounter in males. Higher education was significantly associated with an increased probability of condom use in females. The study shows that condom use was more predicted by sociodemographic factors rather than mobility, multiple sexual partnership, and HIV knowledge. However, as low variance was predicted among females, condom use might be more determined by the willingness of their partners and gender power relation. A condom promotion program should consider social determinants such as gender, age, marital status, ethnicity, education, and economic status. Condom promotion should equally focus on both urban and rural areas. Geographical accessibility of condoms and knowledge of HIV infection might not be sufficient to make people use condoms.

## Figures and Tables

**Table 1:** Sociodemographic Characteristics of the Study Population (*N* = 12,860).

Variables		Number	Percentage/Mean#
Sex	Male	3017	23.4
	Female	9843	76.6
Age group (in years)	15–24	3151	24.9
	25–29	2494	19.1
	30–34	2142	16.8
	35–39	2068	15.9
	40–49	3005	23.3
Ethnicity	Lower caste	1812	14.6
	Others caste	5846	50.9
	Uppers caste	5202	34.5
Religion	Others	802	7.0
	Buddha	1064	8.4
	Hindu	10,994	84.6
Current marital Status	Others	790	6.1
	Married	12070	93.9
Education	No education	5167	41.1
	Primary	2559	20.0
	Secondary	4030	30.9
	Higher	1104	8.0
Occupation	Did not work	2167	18.4
	Skilled/unskilled manual	1368	10.7
	Agriculture	6803	52.6
	Service *	2522	18.3
Wealth quintile	Poorest	2565	17.0
	Poorer	2334	18.7
	Middle	2399	21.0
	Richer	2540	21.3
	Richest	3022	21.9
Ecological region	Mountain	2094	6.5
	Hill	5000	39.8
	Terai	5766	53.7
Development region	Eastern	2971	23.6
	Central	3065	33.9
	Western	2292	20.6
	Mid-western	2445	12.2
	Far-western	2087	9.6
Type of residence	Urban	3639	14.0
	Rural	9221	86.0
Mobility	No	7826	60.2
	Yes	1821	14.0
	Missing	3360	25.8
Multiple sexual partners **	Yes	1627	12.3
HIV knowledge Score	Mean	11,009	7.5
Age at first sex	Mean	12,756	17.8
	Missing	1851	16.8
Condom use ***	No	10,193	79.5
	Yes	1106	7.6
	Missing	1561	12.9

* Professional/technical/managerial; ** 28.7% among currently unmarried and 11.2% among married; *** 16.3% of males and 6.2% of females; #Percentage was adjusted for sample weight, multistage sampling and cluster weight. Thus, the percentage is not equal to unweighted count.

**Table 2:** Condom Use by Sociodemographic and Behavioral Characteristics.

Variables	Categories	Total Sample (*N* = 11,299)				Unmarried Sample (*N* = 284)			
		*n*	%#	χ^2^ Value	*p* Value	*n*	%#	χ^2^ Value	*p* Value
Gender	Male	2837	16.3	268.67	<0.001	245	68.3	30.23	0.000
	Female	8462	6.2			39	20.9		
Age group (in years)	15–24	2780	14.0	182.55	<0.001	215	69.1	NA	NA
	25–29	2187	9.8			39	61.6		
	30–34	1907	7.9			15	30.3		
	35–39	1817	7.0			6	41.7		
	40–49	2608	4.0			9	0.0		
Ethnicity	Lower caste	1593	4.2	75.77	<0.001	32	53.2	1.59	0.581
	Others caste	5084	8.3			146	64.4		
	Uppers caste	4622	11.3			106	62.6		
Religion	Others	680	7.6	1.54	0.764	24	62.3	0.207	0.930
	Buddha	876	8.7			31	66.7		
	Hindu	9743	8.9			229	61.9		
Marital status	Others	284	62.3	1084.91	<0.001	NA	NA	NA	NA
	Married	11,015	7.3			NA	NA		
Education	No schooling	4428	3.2	614.72	<0.001	25	19.8	41.68	<.001
	Primary	2259	5.3			34	35.9		
	Secondary	3590	13.9			145	73.2		
	Higher	1022	24.3			80	70.7		
Occupation	Did not work	1911	10.0	216.76	<0.001	64	63.4	14.08	<0.008
	Manual	1225	9.8			50	58.5		
	Agriculture	5870	5.5			70	46.0		
	Service *	2293	15.8			100	74.4		
Wealth quintile	Poorest	2243	4.3	258.43	<0.001	28	39.5	18.84	0.006
	Poorer	2047	5.7			45	47.5		
	Middle	2093	6.6			47	57.9		
	Richer	2189	9.4			62	80.3		
	Richest	2727	16.1			102	64.8		
Ecological region	Mountain	1872	5.9	18.491	0.018	36	52.9	0.715	0.597
	Hill	4347	10.0			112	61.3		
	Terai	5080	8.2			136	64.0		
Development region	Eastern	2515	8.2	50.406	0.001	63	72.8	10.04	0.058
	Central	2751	7.3			72	58.1		
	Western	1936	8.8			60	63.4		
	Mid-western	2215	9.5			53	43.8		
	Far-western	1882	14.0			36	73.5		
Type of residence	Rural	8043	7.7	93.80	<0.001	182	62.0	0.070	0.068
	Urban	3256	15.0			102	63.9		
Mobility	No	6604	8.8	22.99	<0.001	149	61.3	0.902	0.389
	Yes	1568	12.7			91	67.4		
Multiple sexual partners	No	9779	7.9	72.85	<0.001	137	60.6	0.342	0.621
	Yes	1520	14.6			147	63.9		

*n* = number, % = percentage, χ^2^ chi-square; * Professional/technical/managerial; #Percentage was adjusted for sample weight, multistage sampling and cluster weight. Thus, the percentage is not equal to unweighted count.

**Table 3:** Logistic Regression Analysis of Factors Associated with Condom Use among Males.

Factors	Variables		Adjusted OR (95% CIs)			
			Model 1	Model 2	Model 3	Model 4
Community factors	Ecological region		*p* = 0.011	*p =* 0.147	*p* = 0.099	*p* = 0.037
		Mountain	1	1	1	1
		Hill	1.89 (1.24–2.87)	1.58 (0.96–2.59)	148 (0.85–2.58)	1.54 (0.88–2.69)
		Terai	1.70 (1.11–2.62)	1.30 (0.78–2.16)	1.04 (0.58–1.87)	1.01 (0.55–1.82)
	Development region		*p* = 0.027	*p* < 0.001	*p* < 0.001	*p* < 0.001
		Eastern	1	1	1	1
		Central	0.74 (0.48–1.15)	0.75 (0.47–1.19)	0.81 (0.48–1.38)	0.82 (0.48–1.40)
		Western	1.06 (0.68–1.67)	1.07 (0.66–1.72)	1.48 (0.86–2.54)	1.55 (0.88–2.73)
		Mid-Western	1.08 (0.67–1.74)	1.44 (0.84–2.49)	1.53 (0.82–2.85)	1.63 (0.87–3.06)
		Far-Western	1.61 (0.94–2.76)	2.57 (1.46–4.52)	3.77 (2.08–6.82)	4.28 (2.31–7.93)
	Type of residence		*p* < 0.001	*p* = 0.109	*p =* 0.679	*p =* 0.823
		Rural	1	1	1	1
		Urban	1.98 (1.50–2.61)	1.33 (0.93–1.91)	1.08 (0.73–1.60)	0.95 (0.64–1.41)
Sociodemographic factors	Age group (in years)			*p* < 0.001	*p* < 0.001	*p* < 0.001
		15–24		1	1	1
		25–29		0.67 (0.45–0.99)	0.52 (0.33–0.81)	0.50 (0.32–0.80)
		30–34		0.47 (0.29–0.75)	0.45 (0.26–0.76)	0.45 (0.26–0.77)
		35–39		0.53 (0.34–0.81)	0.43 (0.26–0.70)	0.44 (0.27–0.72)
		40–49		0.32 (0.20–0.51)	0.27 (0.15–0.50)	0.26 (0.14–0.48)
	Ethnicity			*p =* 0.092	*p =* 0.197	*p =* 0.215
		Lower caste		1	1	1
		Others		1.56 (0.94–2.59)	1.60 (0.92–2.77)	1.57 (0.90–2.72)
		Upper caste		1.75 (1.05–2.92)	1.64 (0.93–2.90)	1.63 (0.92–2.89)
	Religion			*p =* 0.945	*p =* 0.774	*p =* 0.527
		Others		1	1	1
		Buddha		1.14 (0.50–2.61)	1.13 (0.46–2.76)	1.17 (0.47–2.92)
		Hindu		1.09 (0.60–1.98)	0.92 (0.47–1.80)	0.85 (0.44–1.66)
	Marital status			*p* < 0.001	*p* < 0.001	*p* < 0.001
		Married		1	1	1
		Others		10.71 (7.28–15.77)	11.88 (7.67–18.36)	12.88 (8.27–20.05)
	Education			*p =* 0.007	*p =* 0.116	*p =* 0.178
		No schooling		1	1	1
		Primary		0.77 (0.39–1.50)	0.81 (0.36–1.83)	0.99 (0.41–2.39)
		Secondary		1.49 (0.85–2.61)	1. 50 (0.73–3.09)	1. 67 (0.77–3.63)
		Higher		1.74 (0.94–3.20)	1.30 (0.60–2.83)	1.39 (0.60–3.19)
	Occupation			*p =* 0.638	*p =* 0.650	*p =* 0.826
		Did not work		1	1	1
		Manual		0.70 (0.31–1.56)	0.78 (0.30–2.03)	0.76 (0.29–2.00)
		Agriculture		0.66 (0.29–1.50)	0.60 (0.22–1.64)	0.67 (0.24–1.87)
		Service *		0.80 (0.36–1.79)	0.78 (0.30–2.04)	0.81 (0.31–2.15)
	Wealth quintile			*p =* 0.003	*p =* 0.001	*p* < 0.001
		Poorest		1	1	1
		Poorer		0.74 (0.43–1.27)	0.64 (0.33–1.22)	0.74 (0.39–1.41)
		Middle		1.18 (0.67–2.08)	1.09 (0.54–2.20)	1.28 (0.65–2.52)
		Richer		1.50 (0.88–2.55)	1.68 (0.91–3.10)	2.10 (1.18–3.74)
		Richest		2.21 (1.19–4.11)	2.41 (1.15–5.06)	3.25 (1.59–6.64)
Behavioral factors	Mobility				*p =* 0.907	*p =* 0.566
		No			1	1
		Yes			1.02 (0.71–1.45)	1.11 (0.77–1.60)
	Multiple sex partners				*p =* 0.743	*p =* 0.916
		No			1	1
		Yes			1.05 (0.78–1.41)	1.01 (0.74–1.38)
	Age at first sex				*p =* 0.015	*p =* 0.009
					1.07 (1.01–1.14)	1.08 (1.02–1.15)
HIV knowledge	HIV knowledge					*p =* 0.163
						0.90 (0.79–1.04)
Cox and Snell *R*^2^			0.018	0.191	0.211	0.220

*p* indicates *p* value and is placed before the reference value for each variable, CIs: confidence intervals, OR: odds ratio; * Professional/technical/managerial.

**Table 4:** Logistic Regression Analysis of Factors Associated with Condom Use among Females.

Factors	Variables			Adjusted OR (95% CIs)		
			Model 1	Model 2	Model 3	Model 4
Community factors	Ecological region		*p =* 0.034	*p =* 0.003	*p =* 0.012	*p* = 0.010
		Mountain	1	1	1	1
		Hill	1.63 (1.08–2.47)	1.22 (0.81–1.82)	1.01 (0.65–1.56)	0.99 (0.63–1.54)
		Terai	1.30 (0.86–1.98)	0.78 (0.51–1.20)	0.66 (0.41–1.07)	0.65 (0.40–1.06)
	Development regions		*p* < 0.001	*p* < 0.001	*p* < 0.001	*p* < 0.001
		Eastern	1	1	1	1
		Central	0.83 (0.56–1.23)	0.80 (0.55–1.15)	0.96 (0.62–1.49)	0.87 (0.58–1.31)
		Western	1.02 (0.69–1.53)	1.02 (0.70–1.49)	1.09 (0.71–1.67)	1.05 (0.68–1.62)
		Mid-western	1.34 (0.90–1.99)	1.92 (1.33–2.78)	1.99 (1.25–3.14)	1.91 (1.21–3.01)
		Far-western	2.21 (1.56–3.12)	3.89 (2.69–5.62)	4.21 (2.64–6.69)	3.83 (2.40–6.10)
	Type of residence		*p* < 0.001	*p =* 0.330	*p =* 0.711	*p* = 0.473
		Rural	1	1	1	1
		Urban	2.23 (1.75–2.84)	1.13 (0.88–1.45)	1.05 (0.80–1.38)	1.10 (0.83–1.47)
Sociodemographic factors	Age group (in years)			*p* < 0.001	*p =* 0.001	*p* = 0.001
		15–24		1	1	1
		25–29		0.90 (0.68–1.19)	0.84 (0.61–1.14)	0.84 (0.61–1.15)
		30–34		0.82 (0.60–1.11)	0.75 (0.52–1.07)	0.71 (0.49–1.04)
		35–39		0.68 (0.46–0.99)	0.65 (0.41–1.03)	0.65 (0.41–1.03)
		40–49		0.41 (0.28–0.61)	0.34 (0.20–0.56)	0.29 (0.16–0.51)
	Ethicality			*p =* 0.013	*p =* 0.149	*p* = 0.071
		Lower caste		1	1	1
		Others		1.95 (1.25–2.06)	1.64 (0.98–2.74)	1.82 (1.08–3.06)
		Upper caste		1.73 (1.13–2.64)	1.58 (0.97–2.56)	1.61 (0.97–2.68)
	Religion			*p =* 0.255	p=0.124	*p* = 0.326
		Other		1	1	1
		Buddha		1.16 (0.59–2.25)	1.10 (0.54–2.25)	1.04 (0.48–2.22)
		Hindu		0.82 (0.50–1.33)	0.71 (0.42–1.22)	0.76 (0.41–1.41)
	Marital status			*p =* 0.037	*p =* 0.132	*p =* 0.078
		Married		1	1	1
		Others		2.96 (1.06–8.26)	2.29 (0.77–6.81)	2.85 (0.88–9.25)
	Education			*p* < 0.001	*p* < 0.001	*p* = 0.001
		No schooling		1	1	1
		Primary		1.25 (0.90–1.73)	1.07 (0.73–1.58)	1.00 (0.67–1.49)
		Secondary		2.41 (1.71–3.41)	1.93 (1.30–2.87)	1.66 (1.14–2.42)
		Higher		3.96 (2.66–5.88)	2.76 (1.70–4.49)	2.57 (1.56–4.24)
	Occupation			*p =* 0.833	*p =* 0.998	*p* = 0.999
		Did not work		1	1	1
		Manual		0. 86 (0.42–1.76)	0.98 (0.41–2.29)	0.98 (0.45–2.10)
		Agriculture		0.90 (0.67–1.19)	0.97 (0.70–1.33)	1.01 (0.71–1.42)
		Service *		0.88 (0.66–1.17)	0.98 (0.71–1.36)	1.01 (0.73–1.41)
	Wealth quintile			*p* < 000	*p* < 0.001	*p* < 0.001
		Poorest		1	1	1
		Poorer		1.97 (1.31–2.98)	2.25 (1.43–3.52)	1.99 (1.27–3.11)
		Middle		2.09 (1.34–3.27)	2.28 (1.34–3.88)	2.19 (1.25–3.83)
		Richer		2.61 (1.63–4.18)	3.20 (1.87–5.46)	2.83 (1.63–4.90)
		Richest		4.31 (2.55–7.28)	5.51 (3.09–9.82)	4.83 (2.63–8.87)
Behavioral factors	Mobility				*p* = 0.938	*p* = 0.989
		No			1	1
		Yes			0.98 (0.67–1.42)	0.99 (0.68–1.45)
	Multiple sex partners				*p* = 0.908	*p* = 0.967
		No			1	1
		Yes			0.95 (0.45–2.03)	0.98 (0.45–2.11)
	Age at first sex				*p* = 0.186	*p* = 0.229
					1.03 (0.98–1.07)	1.02 (0.97–1.07)
Knowledge	HIV knowledge					*p* = 0.554
						1.03 (0.92–1.15)
Cox and Snell *R^2^*			0.012	0.053	0.055	0.054

*p* indicates *p* value and is placed before the reference value for each variable, CIs: confidence intervals, ORs: odds ratios; * Professional/technical/managerial.

## Appendix A

**Table A1:** Assessment of HIV Knowledge.

Number of Questions	Questions Asked	Coding
1	Have you heard of about other infections that can be transmitted through sexual contact (STI)?	Yes = 1 No = 0
2	Have you ever heard of an illness called AIDS?	Yes = 1 No = 0
3	Can people reduce their chances of acquiring the AIDS virus by having just one uninfected sex partner who has no other sex partners?	Yes = 1 No/Do not know = 0
4	Can people reduce their chances of acquiring the AIDS virus by using a condom every time they have sex?	Yes = 1 No/Do not know = 0
5	Can people acquire the AIDS virus from mosquito bites?	No = 1 Yes/Do not know = 0
6	Is it possible for a healthy looking person to have the AIDS virus?	Yes = 1 No/Do not know = 0
7	Can one get HIV by sharing food with a person who has AIDS?	No = 1 Yes/Do not know = 0
8	Can HIV be transmitted from a mother to her baby during delivery?	Yes = 1 No/Do not know = 0
9	Can HIV be transmitted from a mother to her baby by breastfeeding?	Yes = 1 No/Do not know = 0
10	Are there any special drugs that a doctor or nurse can give to a woman infected with the AIDS virus to reduce the risk of transmission to the baby?	Yes = 1 No/Do not know = 0
